# Distribution pattern of mesangial C4d deposits as predictor of kidney failure in IgA nephropathy

**DOI:** 10.1371/journal.pone.0252638

**Published:** 2021-06-03

**Authors:** Suchin Worawichawong, Sirithep Plumworasawat, Wisit Liwlompaisan, Vasant Sumethkul, Bunyong Phakdeekitcharoen, Umaporn Udomsubpayakul, Panus Chalermsanyakorn, Chagriya Kitiyakara

**Affiliations:** 1 Department of Pathology, Faculty of Medicine Ramathibodi Hospital, Mahidol University, Bangkok, Thailand; 2 Department of Medicine, Faculty of Medicine Ramathibodi Hospital, Mahidol University, Bangkok, Thailand; 3 Section for Clinical Epidemiology and Biostatistics, Faculty of Medicine Ramathibodi Hospital, Mahidol University, Bangkok, Thailand; University of KwaZulu-Natal, SOUTH AFRICA

## Abstract

Mesangial C4d deposits have been associated with worse outcomes in Western patients with IgA nephropathy (IgAN), but there is limited data in Asians. Previously, a high proportion of stained glomeruli was often required for the classification of C4d positive (C4d+ve). Positive staining in lower proportion of staining would be classified as C4d-ve. This retrospective study evaluated the prognostic value of C4d+ve using a less stringent definition (one C4d+ve glomerulus) in Thai patients with IgAN (n = 120). Baseline findings and outcomes were compared between those with more extensive C4d staining patterns and those with more restricted staining. Clinico-pathologic parameters and risk for kidney outcomes (kidney failure or decline GFR50%) were compared between C4d+ve versus C4d-ve, and between different patterns: Focal (< 50%) versus Diffuse (≥ 50% of glomeruli); or Global (≥ 50) versus Segmental (< 50% of mesangial area). The hazard ratios were estimated using Cox proportional hazard models for Model 1 (Oxford score+ C4d) and Model 2 (Model 1+ clinical factors). C4d+ve (n = 81) had lower eGFR, more global sclerosis, and interstitial fibrosis than C4d-ve at baseline. The 5-year kidney survival for C4d+ve was lower (53.7%) than C4d-ve (89.7%); P = 0.0255. By univariate analysis, T1, T2, C4d+ve, eGFR<60, proteinuria were predictors of kidney outcome. By multivariate analysis, proteinuria, T1, T2 and C4d+ve were independent predictors (Model 2 HR (95% CI) C4d+ve: 3.24 (1.09–9.58), p = 0.034). Segmental had lower eGFR, higher tubulointerstitial fibrosis, and segmental sclerosis compared to Global pattern. Clinicopathological parameters were not different between Focal and Diffuse patterns. Outcomes were similar between staining patterns. In conclusion, C4d staining may be a valuable marker of poor prognosis in Asian patients with IgAN. Less stringent criteria for C4d+ve should be considered as no differences in outcomes were observed between more extensive staining with less extensive patterns. More studies are needed to identify the optimum criteria for C4d+ve.

## Introduction

IgA nephropathy (IgAN) is the most common primary glomerular disease worldwide with Asian countries having among the highest prevalence [1]. IgAN is defined by the predominant deposition of IgA in the glomerular mesangium, and is characterized by a highly variable course ranging from asymptomatic patients to those with progressive deterioration of kidney function to kidney failure). Several clinical factors including hypertension, proteinuria, low baseline kidney function, and adverse kidney histological features are associated with poor outcome in IgAN [2, 3]. The Oxford classification of the histopathological findings has been widely used to predict outcomes in IgAN patients. In 2016, the classification system has been updated to include the percent of crescents [4]. Nonetheless, current clinicopathologic parameters cannot fully predict the progression of IgAN. New prognostic markers may be useful to guide treatment of IgAN.

The pathogenesis of IgAN is believed to involve a ‘multi-hit’ process. Mesangial deposition of abnormal galactose-deficient IgA1, and immune complexes induce mesangial cell proliferation, increased matrix synthesis, and kidney cell injury [5]. Several studies have demonstrated that activation of the complement system promotes the inflammatory cascade and potentiates tissue injury in IgAN [6, 7]. Mesangial IgA deposits can activate complement via two pathways. Mesangial IgA deposits colocalized with C3 but not with IgG or C1q deposits in a majority of patients, suggesting that the alternative pathway is the major complement pathway activated. Mesangial mannose binding lectin (MBL) deposits were found to be colocalized with IgA, C3 and C4d in about 30% of IgAN patients. This is in keeping with complement activation by the lectin pathway, which has been associated with more severe proteinuria, kidney damage, and reduction in GFR at diagnosis [8, 9].

C4d is a product of C4 cleavage that covalently binds to surrounding molecules acting as a long-lasting marker of complement activation by the lectin pathway in IgAN [10]. Mesangial C4d deposits has been shown to be an independent predictor for kidney function decline in recent studies performed in mostly European populations [11–14]. At present, the value of C4d deposits as predictors of outcome in IgAN remains unclear for many reasons. First, the optimal criteria for diagnosis of C4d+ve is unknown. The criteria used to classify patients as C4d positive (C4d+ve) or C4d negative (C4d-ve) were chosen arbitrarily, ranging from any positive glomeruli [13] to 75% in different studies [11]. Most studies that have evaluated the long term kidney endpoints have often used 50% or greater glomerular involvement, which corresponds to a diffuse staining pattern, to define C4d+ve. The clinical significance of lower proportion of glomeruli involved by C4d staining, corresponding to a focal staining pattern, is unclear. Moreover, there have been very limited information evaluating the outcomes between those with staining of parts of the glomeruli (segmental) versus those with whole glomerular surface staining (global). Finally, there is still limited data on the long term prognostic value of C4d staining in Asian adults despite Asians having high prevalence of IgAN.

Similar to other Asian countries, IgAN nephropathy is the commonest cause of kidney failure due to primary glomerular disease in Thailand (http://www.nephrothai.org). This study aimed to evaluate the long term kidney prognosis of C4d+ve patients using a less stringent criteria of at least one glomerulus being Cd4+ve and to evaluate the clinicopatholgical presentations and prognosis of different patterns of C4d glomerular staining in Thai patients with IgAN.

## Materials and methods

This retrospective cohort study included patients, who attended the nephrology outpatient clinic, Ramathibodi Hospital with IgAN confirmed by kidney biopsy performed during 2006 to 2012 with at least five glomeruli in the paraffin-embedded section. Patients with Henoch–Schönlein purpura, liver diseases, diabetes, systemic diseases, and other secondary IgAN were excluded.

Medical records between 2006 to 2015 were reviewed. Demographic, clinical information, and laboratory results were recorded at the time of kidney biopsy as the baseline. Patients were managed in the outpatient clinic by nephrologists according to standard guidelines [15]. Serum creatinine and need for dialysis was recorded for each visit. The estimated glomerular filtration rate (eGFR) was calculated from serum creatinine by CKD-EPI formula [16]. Hypertension was defined as more than 140/90 mmHg or use of antihypertensive medications [17].

The study was performed in accordance with the Declaration of Helsinki and was approved by Committee on Human Rights Related to Research Involving Human Subjects, Faculty of Medicine Ramathibodi Hospital, Mahidol University, Bangkok, Thailand. The ethics committee waived the requirement for informed consent since this study used routinely acquired clinical information in a retrospective manner, and results were reported as anonymized group and individual patient data was not shown.

### Histopathologic analyses

#### IgA diagnosis and Oxford classification

Kidney biopsy was performed by nephrologists for diagnostic purposes according to routine clinical indications. Pathology technicians immediately processed the tissues and routinely stored the kidney tissues as de-identified paraffin-embedded blocks. Paraffin-embedded kidney tissues (2 μm sections) were stained with hematoxylin-eosin, Masson trichrome, periodic acid–Schiff, and Jones methenamine silver stains. Frozen sections were stained with antibodies against IgG, IgA, IgM, C1q, and C3 (dilution 1:60) by immunofluorescence (Dako, Glostrup, Denmark, Catalog# F 0202, F 0203, F0204, F 0201, F 0254).

IgAN was defined by the presence of IgA mesangial deposits as dominant or codominant Ig. Histologic findings were graded according to the Oxford classification (MEST+C) using the updated 2016 criteria [4]. Five variables were scored as follows: mesangial hypercellularity (M0:<50%;M1:≥ 50% of glomeruli), endocapillary hypercellularity (E0: absent; E1: any glomeruli showing endothelial hypercellularity), segmental glomerulosclerosis (S0:absent; S1: any glomeruli showing segmental sclerosis), and tubular atrophy/interstitial fibrosis (T0:0–25%;T1: 26%–50%; and T2: >.50% of cortical area). Cellular or fibrocellular crescent (C0: absent; C1:0–25%; C2≥25% of glomeruli).

#### C4d staining and scoring

C4d immunohistochemical staining was performed on 3-μm-sectioning formalin fixed paraffin embedded tissue by pathology technicians using rabbit monoclonal anti-human C4d (Cell Marque^®^, SP91 clone, Rocklin, CA, US; catalog # 404R-15) stained on VENTANA BenchMark machine with ultraView Universal DAB detection protocol. CC1 solution was used as standard pretreatment for 32 min. Primary monoclonal anti-C4d antibody was applied at dilution 1: 200 and incubated at 37 C for 32 min. Antibody-mediated rejected kidney allograft and normal kidney tissues were used as positive and negative controls, respectively.

The scoring of C4d staining into negative or positive was performed independently by two pathologists (SW and PS). Disagreements were resolved by consensus after discussion. C4d+ve was defined as at least one nonsclerotic glomerulus was positive for C4d in mesangial cells involving more than 10% of glomerulus.

**Fig 1:**
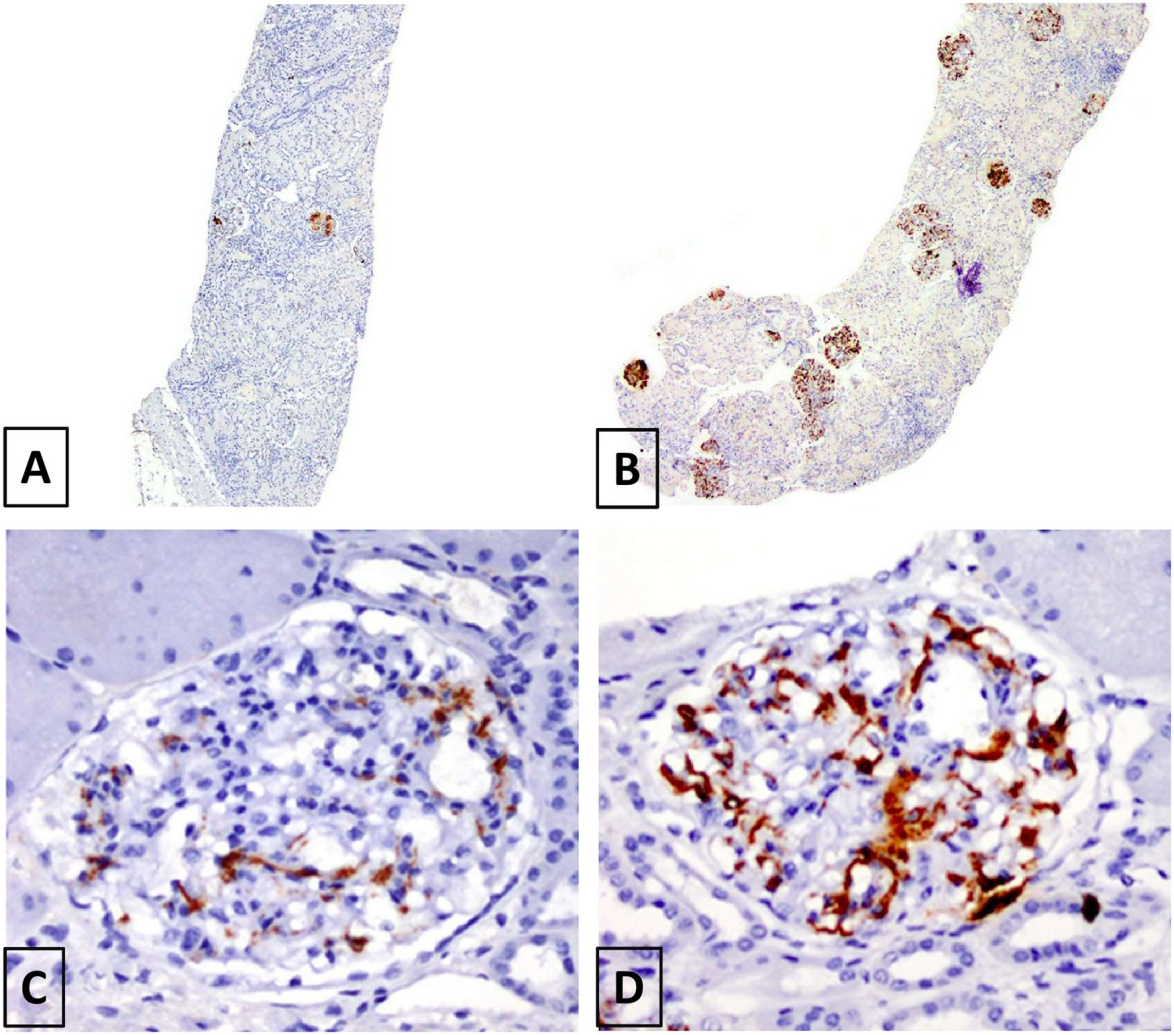
Patterns of mesangial C4d staining. (A) Focal, (B) Diffuse, (C) Segmental and (D) Global by anti-C4d antibody immunoperoxidase staining.

The pattern of C4d+ve staining was classified into 1) Focal (staining affected less than 50% of total glomeruli) or Diffuse (staining affected more than 50% of total glomeruli), and 2) Global (staining affecting more than 50% of mesangial area) or Segmental (staining affected less than 50% of mesangial area) (Fig 1). This criteria is modified from the International Society of Nephrology/Renal Pathology Society (ISN/RPS) 2003 classification of lupus nephritis such that C4d staining was classified as segmental if >50% of the C4d stained glomeruli had segmental staining, and global when >50% of the stained glomeruli had global staining [18].

### Outcomes

The primary outcome is the combined adverse kidney outcome: Kidney Failure (the need for kidney replacement therapy or eGFR <15 ml/min/1.73m2) or a decline of eGFR by more than 50% (ΔGFR50%). The secondary outcome was Kidney failure.

### Statistical analysis

Variables with normal distribution are presented as mean ± standard deviation, non-normal distribution as median (25,75 percentile) and categorical variables as percentages. Between group comparisons were performed using the chi-squared/Fisher test for categorical variables or using the Student’s t-test or non-parametric test for continuous variables, as appropriate. Missing data were excluded.

Kaplan–Meier analyses were performed to evaluate the effects of C4d on kidney outcomes after excluding patients with eGFR<15ml/min/1.73m^2^ at baseline. Survival time for each patient was computed from baseline evaluation to the last follow-up or the outcomes of interest. Univariate survival comparisons were made using the log-rank test. The Cox proportional hazards model was used to estimate the hazard ratios of each parameter with regards to kidney outcomes using 2 models of adjustments: Model 1, Pathological (Oxford score + C4d); and Model 2, Pathological + baseline clinical parameters (eGFR, proteinuria, hypertension). Variables were selected by backward elimination using likelihood ratio tests. Statistical software STATA/SE version 15.1 software were used for data analysis. A two-sided P<0.05 indicated statistical significance.

## Results

### Patient characteristics

During 2006 to 2012, 131 patients were diagnosed with idiopathic IgA nephropathy by kidney biopsy. Eleven patients were excluded due to insufficient kidney tissue for evaluation leaving 120 patients for analysis.

#### Baseline characteristics by positive or negative C4d staining

The baseline clinicopathological data are shown in Table 1. Positive C4d glomerular mesangial staining was observed in 81 of 120 (67.5%) patients. C4d+ve patients had lower eGFR, more global sclerosis, and interstitial fibrosis compared to C4d-ve patients.

**Table 1:** Clinicopathological data at the time of kidney biopsy and outcomes at follow-up by positive or negative C4d staining.

	All	C4d positive	C4d negative	P-value
Baseline Characteristics				
All patients N	120	81	39	
Age (Years)	37.3 ± 12.6	38.5 ± 12.2	34.8 ± 13.4	0.133
Male	46.7%	43.2%	53.8%	0.274
Follow up time (months)	51.6 ± 17.1	48.3 ± 19.7	57.6 ± 8.5	**0.007**
Systolic BP (mmHg)	132.5 ± 17.3	133.4 ± 17.8	131.0 ± 16.4	0.499
Diastolic BP (mmHg)	82.0 ± 11.3	82.0 ± 11.6	82.1 ± 10.9	0.971
Hypertension	45.8%	45.7%	46.2%	0.961
Proteinuria (g/24hr)	2.24 ± 1.98	2.42 ± 2.09	1.94 ± 1.75	0.249
Proteinuria >1 g/day (%)	74.8%	76.6%	71.1%	0.518
Heavy proteinuria ≥ 3 g/day (%)	25.2%	29.9%	15.8%	0.102
Serum creatinine (mg/dL)	1.58 ± 0.86	1.69 ± 0.88	1.37 ± 0.80	0.069
eGFR (mL/min/1.73 m^2^)	64 ± 41	55. ± 38	81 ± 40	**0.001**
Mesangial proliferation (M1)	93.3%	93.8%	92.3%	0.714
Endocapillary proliferation (E1)	33.3%	34.6%	30.8%	0.679
Segmental sclerosis(S1)	63.3%	67.9%	53.8%	0.135
Interstitial fibrosis/ Tubular atrophy (T1-T2)	49.2%	55.6%	35.9%	**0.044**
Crescent C1 C1:0–25%;	14.6%	13.6%	16.2%	0.722
Crescent C2 C2≥25%	8.7%	10.6%	5.4%	0.484
Crescent (C1 or C2)	23.3%	24.2%	21.6%	0.763
Global sclerosis	80.8%	87.7%	66.7%	**0.006**
Arterial intimal fibrosis (Moderate to severe)	28.2%	30.8%	23.1%	0.383
Hyaline deposit in arteriole (Moderate to severe)	30.0%	33.3%	23.1%	0.251
Outcomes				
Patients with follow-up and no KF at baseline N	101	65	36	
KF or ΔGFR50% N	26 (25.7%)	21 (32.3%)	5 (13.9%)	**0.043**
ΔGFR50% N	25 (24.8%)	20 (30.8%)	5 (13.9%)	0.060
KF N	20 (19.8%)	17 (26.2%)	3 (8.3%)	**0.031**

eGFR, estimated glomerular filtration rate; KF, Kidney failure or eGFR<15 ml/min/1.73m^2^).

ΔGFR50%, decline in eGFR by more than 50% from baseline.

#### Baseline characteristics according to C4d staining pattern

C4d staining was diffuse in 50 out of 81 C4d+ve patients (62%), and focal in 31 patients (Table 2). Focal pattern tended to be older, more likely to be hypertensive, although this did not reach statistical significance. There were no other differences in the between focal and diffuse patterns.

**Table 2:** Clinicopathological data at the time of kidney biopsy and outcomes at follow-up by patterns of glomerular mesangial C4d staining.

	Patterns of C4d mesangial staining					
	Diffuse	Focal	P-value	Global	Segmental	P-value
Baseline Characteristics						
All C4d +ve N	50	31		44	37	
Age (Years)	36.6 ± 11.9	41.6 ± 12.2	0.069	37.0 ± 12.7	40.2 ± 11.4	0.237
Male (%)	40%	48.4%	0.459	47.7%	37.8%	0.371
Follow up time (months)	49.5 ± 19.8	45.6 ± 19.9	0.530	46.6 ± 20.7	50.4 ± 18.5	0.524
Systolic BP (mmHg)	135.5 ± 17.9	137.6 ± 20.6	0.635	140.1 ± 19.2	131.8 ± 17.6	**0.048**
Diastolic BP (mmHg)	82.7 ± 11.4	84.1 ± 10.8	0.576	83.6 ± 10.8	83.3 ± 11.7	0.965
Hypertension	38%	58.1	0.078	52.3%	37.8%	0.194
Proteinuria (g/24hr)	2.67 ± 2.40	2.35 ± 1.43	0.476	2.83 ± 2.03	2.22 ± 2.13	0.204
Proteinuria >1 g/day (%)	75.0%	79.3%	0.665	83.3%	68.6%	0.128
Heavy Proteinuria ≥3g/day (%)	22.2%	40.7%	0.094	35.0%	21.9%	0.223
Serum creatinine (mg/dL)	1.65±0.88	1.76±0.88	0.633	1.88±0.93	1.52±0.81	0.101
eGFR (mL/min/1.73 m^2^)	56±40	53 ±35	0.711	45±36	68±37	**0.005**
Mesangial proliferation (M1)	96%	90.3%	0.302	90.9%	97.3%	0.234
Endocapillary proliferation (E1)	34%	35.5%	0.891	29.5%	40.5%	0.300
Segmental sclerosis(S1)	74%	58.1%	0.135	79.5%	54.1%	**0.014**
Interstitial fibrosis/ Tubular atrophy (T1-T2)	54%	58.1%	0.720	68.2%	40.5%	**0.013**
Crescent C1 C1:0–25%;	14.6%	12.0%	1.000	6.3%	20.6%	0.151
Crescent C2 C2≥25% of glomeruli	9.8%	12.0%	1.000	12.5%	8.8%	0.705
Crescent	24.4%	24.0%	0.971	18.8%	29.4%	0.312
Global sclerosis	86%	90.3%	0.565	90.9%	83.8%	0.332
Arterial intimal fibrosis (Moderate to severe)	24.5%	41.4%	0.118	38.1%	22.2%	0.130
Hyaline deposit in arteriole (Moderate to severe)	30%	38.7%	0.419	34.09%	32.4%	0.875
Outcomes						
Patients with follow-up and no KF at baseline N	41	24		32	33	
KF (dialysis or GFR <15) or ΔGFR >50% N	12 (29.3%)	9 (37.5%)	0.493	10 (31.3%)	11 (33.3%)	0.857
ΔGFR >50% only N	11 (26.8%)	9 (37.5%)	0.368	9 (28.1%)	11 (33.3%)	0.649
KF (dialysis or GFR<15% only)N	10 (24.4%)	7 (29.2%)	0.672	9 (28.1%)	8 (24.2%)	0.722

eGFR, estimated glomerular filtration rate; KF, Kidney Failure (dialysis or eGFR<15 ml/min/1.73m^2^).

ΔGFR50%, decline in eGFR by more than 50% from baseline.

C4d staining was segmental in 37 patients (46% of C4d+ve) and global in 44 patients. Baseline eGFR was lower in patients with global compared to segmental pattern. The proportion with segmental sclerosis and interstitial fibrosis (T1-T2) was higher in global pattern (Table 2).

### Multivariate analysis of primary kidney outcome (Kidney Failure or ΔGFR50%)

Fourteen patients with eGFR<15ml/min/1.73m^2^ at baseline, and 5 patients who were referred to another hospital after the kidney biopsy were excluded, leaving 101 patients for analysis of outcomes. The follow-up was 51.6 ± 17.1 months.

#### Positive versus negative C4d staining

**Fig 2:**
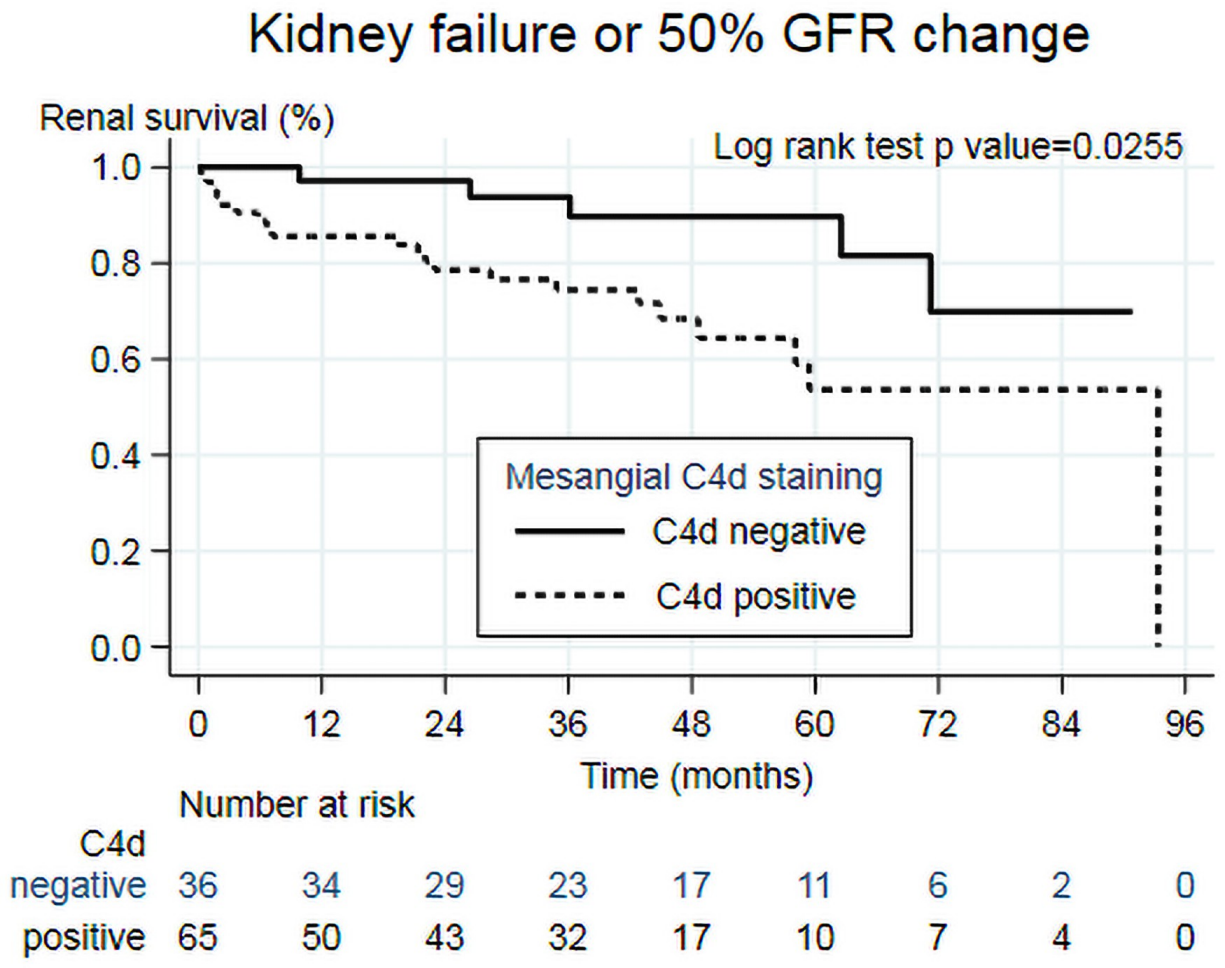
Kaplan-Meier analysis of mesangial C4d staining (positive vs negative) on primary outcome (Kidney Failure or ΔGFR50%).

Overall, 26 (25.7%) of subjects reached primary combined outcome. The median time to event was 40.9 (22.5, 57.1) months. 21 (32.3%) of C4d+ve and 5 (13.9%) of C4d-ve reached end-point (Fig 2). The 5-year outcome-specific survival for patients with C4d+ve was lower (53.7%) than for patients with C4d-ve (89.7%; P = 0.0255).

By univariate analysis, T1, T2, C4d+ve, eGFR<60, heavy proteinuria (≥3g/day) were predictors of primary outcome (Table 3). By multivariate analysis using model 2, heavy proteinuria (≥3g/day), T1, T2 and C4d+ve were predictors of primary outcome (The Model 2 adjusted HR (95% CI) C4d+ve: 3.24 (1.09–9.58), p = 0.034). Substitution of heavy proteinuria (>3g/day) with proteinuria ≥1g vs < 1g/day) did not alter the multivariate results.

**Table 3:** Multivariate analysis of clinicopathological parameters on primary outcome (Kidney Failure or ΔGFR50%).

N = 101	Univariate HR (95%CI)	P Value	Model 1 HR (95%CI)	P Value	Model 2HR (95%CI)	P Value
*Pathological parameters*						
Mesangial hypercellularity						
M0	reference					
M1	1.52 (0.20–11.25)	0.682				
Endocapillary hypercellularity						
E0	reference					
E1	1.54 (0.70–3.41)	0.282				
Segmental glomerulosclerosis						
S0	reference					
S1	2.22 (0.88–5.59)	0.091				
Tubular atrophy/interstitial fibrosis						
T0	reference					
T1	**7.28 (2.68–19.79)**	**<0.001**	**6.79 (2.51–18.40)**	**<0.001**	**6.04 (2.20–16.59)**	**<0.001**
T2	**5.06 (1.69–15.10)**	**0.004**	**5.37 (1.79–16.07)**	**0.003**	**3.68 (1.15–11.80)**	**0.028**
Crescents						
C0	reference					
C1 +C2	1.77 (0.76–4.13)	0.185				
C4d						
Negative	reference					
Positive	**2.92 (1.09–7.81)**	**0.033**	**2.78 (1.03–7.50)**	**0.043**	**3.24 (1.09–9.58)**	**0.034**
*Baseline clinical parameters*						
eGFR						
eGFR > = 60	**reference**					
eGFR <60	**5.60 (2.22–14.10)**	**<0.001**				
Proteinuria						
Absent—Proteinuria <3g/day	**Reference**					
Heavy Proteinuria ≥3g/day	**3.71 (1.71–8.05)**	**0.001**			**2.49 (1.09–5.70)**	**0.030**
Hypertension						
Absent	reference					
Present	1.20 (0.54–2.64)	0.658				

eGFR, estimated glomerular filtration rate (ml/min/1.73m^2^); Kidney Failure, (dialysis or eGFR<15 ml/min/1.73m^2^); ΔGFR50%, decline in eGFR by more than 50% from baseline.

Multivariate Model1: Pathology parameters (Oxford MEST-C score and C4d). Model 2: Pathology + Baseline Clinical parameters (Hypertension, Proteinuria, eGFR).

**Fig 3:**
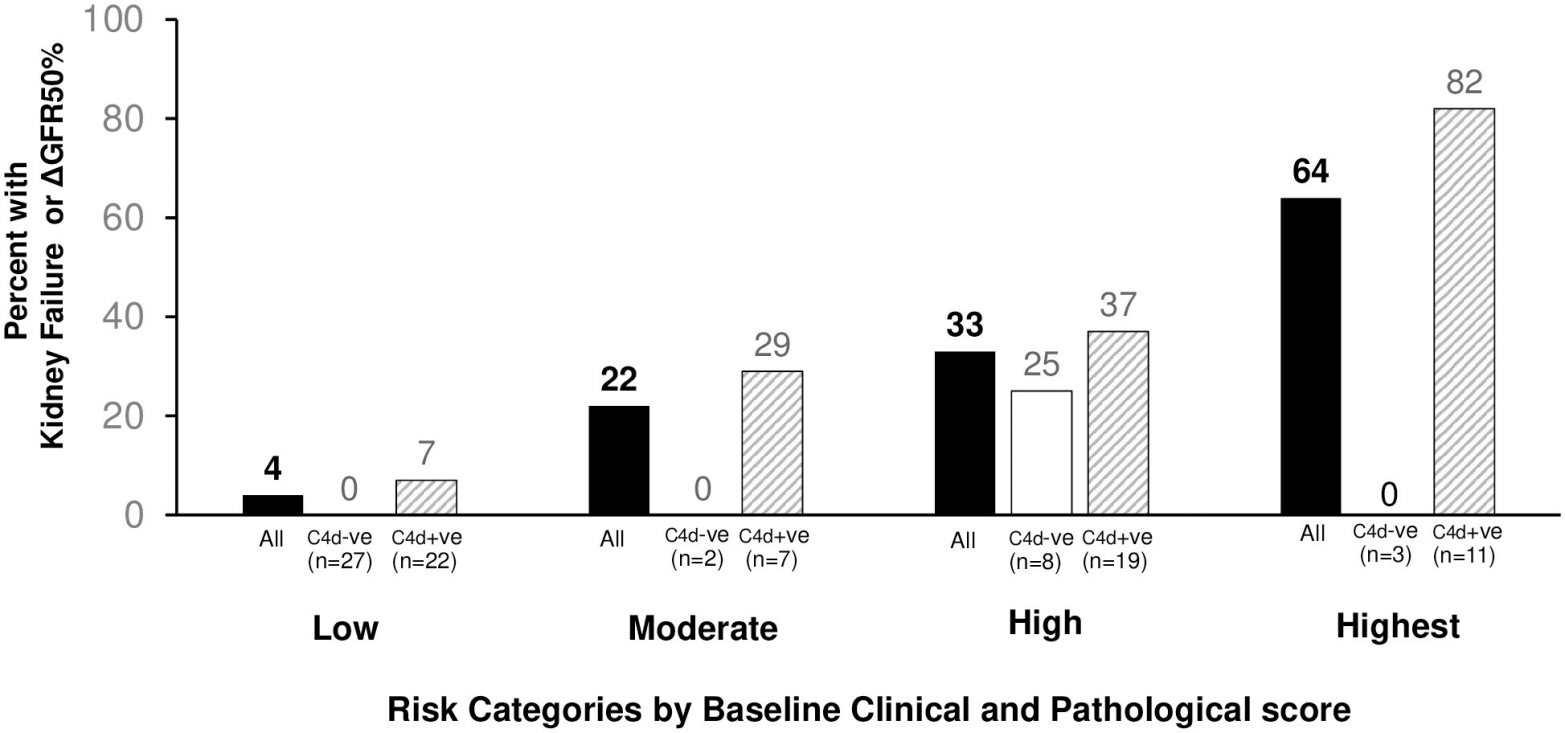
Proportion reaching primary outcome (Kidney Failure or ΔGFR50%) at 5 years according to predicted risk categories and C4d staining. Patients were divided into categories of risk using a model derived from clinical and pathological parameters at baseline: Low (n = 49), Medium (n = 9), High (n = 27), Highest (n = 14). The proportion of patients in each subset reaching primary outcome within 5 years are shown: All in each risk category (black columns); subset in each category stained positive (striped columns) or negative (white column) for C4d.

*Effects of C4d positive in different risk groups on primary outcome at 5 years*. We evaluated the prognostic impact of positive or negative C4d staining in different risk groups based on baseline parameters. We calculated a risk score to reach primary end-point for each patient using a predictive model derived from clinical and MEST+C parameters, which were significant by univariate analysis in Table 3. For this model, we did not include C4d staining, and we combined T1 and T2 into one group (T1+T2) since there is an overlap of the risk ratios compared to T0. Two patients were excluded as they were missing protein level data at baseline. Patients (n = 99) were categorized into quartiles of the predicted risk score calculated using the multivariate model (Final Model: Risk = 0.901 (heavy Proteinuria≥3g/day) + 1.647(tubular atrophy/interstitial fibrosis score T1+T2)). Fig 3 shows that the actual proportion of patients who reached end-point within 5 years from baseline increased progressively from the lowest to the highest predicted risk group. The proportion of patients who reached primary end-point was higher in C4d+ve patients when compared to C4d-ve in all risk categories. The difference in primary outcome between C4d+ve patients versus C4d-ve was greatest in the highest predicted risk category. Analysis of the data for developing primary end-point at 3 years showed similar trends (data not shown).

#### Effects of C4d staining patterns

*Diffuse versus focal*. 12 of 50 with diffuse and 9 of 31 with focal pattern reached end-point. By univariate analysis, there was no difference between focal versus diffuse (HR Focal: 1 (Reference) vs Diffuse: 0.802 (0.33–1.94), p = 0.624) (S1 Fig).

*Global versus segmental*. 10 of 44 with global and 11 of 37 with segmental pattern reached end-point. By univariate analysis, there was no difference between global versus segmental (HR Segmental:1 (Reference) vs Global: 1.19 (0.49–2.85), p = 0.702) (S2 Fig).

*Combined pattern*. The prognosis of combinations of global vs segmental and diffuse vs focal was explored. The proportions of patients reaching end-point for each combination were: segmental +focal, 6/14 (42.8%); segmental +diffuse, 5/19 (26.3%); global+focal, 3/10 (30%); and global +diffuse, 7/22 (33.8%). By univariate analysis, there was no differences in reaching end-point between different combinations (p = 0.87).

### Secondary outcome (Kidney Failure)

**Fig 4:**
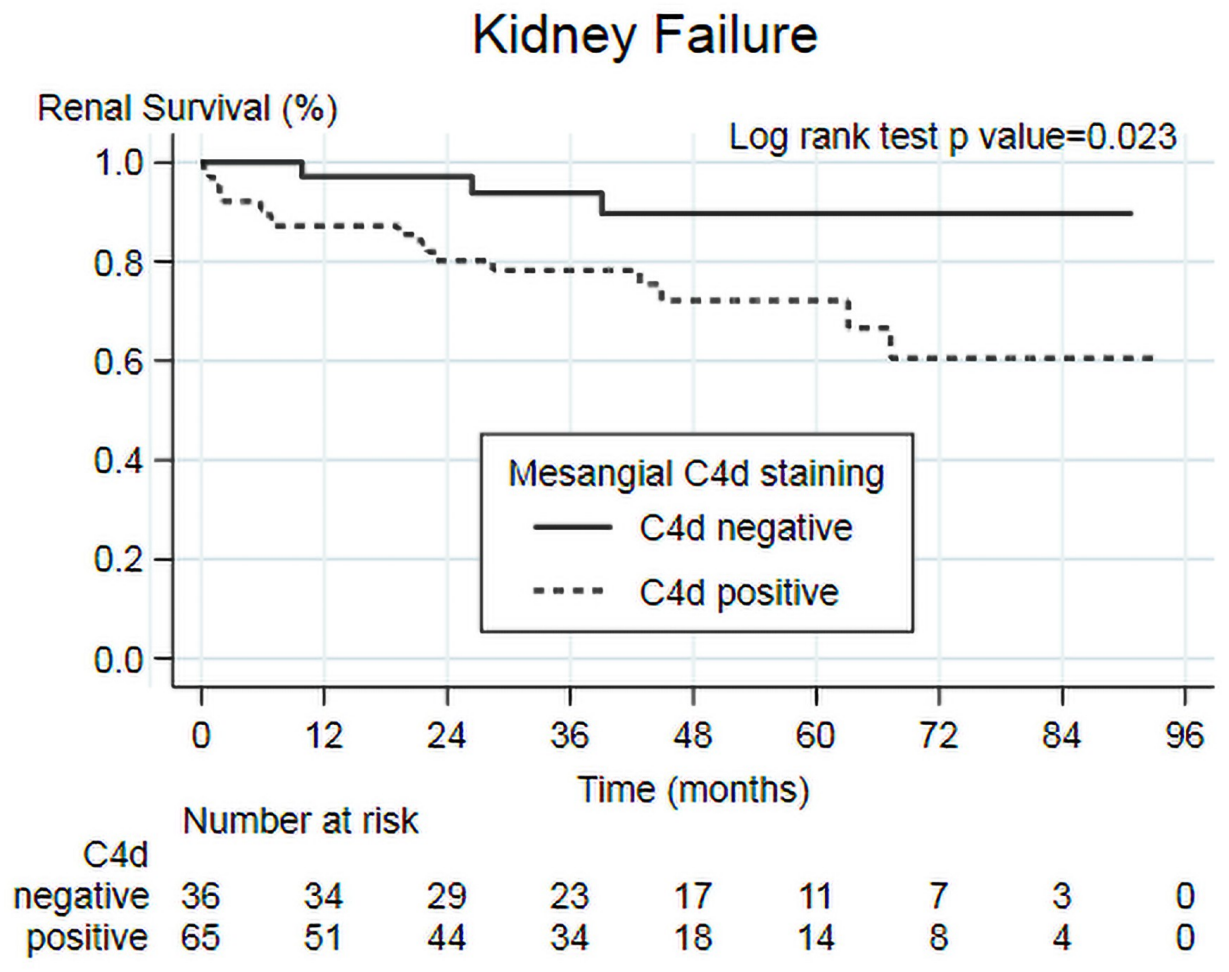
Kaplan-Meier analysis of mesangial C4d staining (positive vs negative) on secondary outcome (Kidney Failure).

Of 101 patients without Kidney Failure at baseline, 22 (20.0%) of subjects reached Kidney Failure. The median time to event was 41.2 (23.0, 60.2) months. 17 (26.2%) of C4d+ve and 3 (8.3%) of C4d-ve reached end-point (Fig 4). The 5-year outcome-specific survival for patients with C4d+ve was lower (72.2%) than for patients with C4d-ve (89.7%; P = 0.023).

By univariate analysis, T1, T2, C4d+ve, eGFR<60, heavy proteinuria ≥3g/day were predictors of Kidney Failure (Table 4). By multivariate analysis model 2, T1, Crescents (C1+C2), C4d+ve, heavy proteinuria ≥3g/day, eGFR<60 were predictors of Kidney Failure (The Model 2 adjusted HR (95% CI) C4d+ve: 9.58 (1.92–47.76), p = 0.006).

**Table 4:** Multivariate analysis of clinicopathological parameters on secondary outcome (Kidney Failure).

N = 101	Univariate HR (95% CI)	P Value	Model 1HR (95% CI)	P Value	Model 2 HR (95% CI)	P Value
*Pathological parameters*						
Mesangial hypercellularity						
M0	reference					
M1	1.202 (0.161–8.99)	0.858				
Endocapillary hypercellularity						
E0	reference					
E1	1.52 (0.63–3.66)	0.356				
Segmental glomerulosclerosis						
S0	reference					
S1	1.95 (0.71–5.38)	0.198				
Tubular atrophy/interstitial fibrosis						
T0	reference					
T1	**9.84 (2.70–35.89)**	**<0.001**	**9.042 (2.48–33.00)**	**0.001**	**5.67 (1.45–22.23)**	**0.013**
T2	**10.50 (2.71–40.70)**	**<0.001**	**10.88 (2.79–42.40)**	**0.001**	**1.72 (0.40–7.31)**	**0.462**
C4d						
Negative	reference					
Positive	**3.75 (1.10–12.82)**	**0.035**	**3.51 (1.023–12.107)**	**0.046**	**9.58 (1.92–47.76)**	**0.006**
Crescent						
C0	reference					
C1 +C2	1.91 (0.76–4.83)	0.169			**3.73 (1.26–11.04)**	**0.017**
*Baseline clinical parameters*						
eGFR						
eGFR ≥60	reference					
eGFR <60	**32.64 (4.35–244.8)**	**0.001**			**46.12 (4.78–444.64)**	**0.001**
Proteinuria						
Absent—Proteinuria <3g/day	reference					
Heavy Proteinuria ≥3g/day	**3.70 (1.57–8.75)**	**0.003**			**5.90 (1.89–18.39)**	**0.002**
Hypertension						
Absent	reference					
Present	1.56 (0.65–3.74)	0.325				

eGFR, estimated glomerular filtration rate (ml/min/1.73m^2^; Kidney Failure, (dialysis or eGFR<15 ml/min/1.73m^2^); ΔGFR50%, decline in eGFR by more than 50% from baseline.

Multivariate Model 1: Pathology parameters (Oxford MEST-C score and C4d).

Model 2: Pathology + Baseline Clinical parameters (Hypertension, Proteinuria, eGFR).

## Discussion

We showed that C4d+ve patients defined as positive mesangial C4d in at least one glomerulus were more likely to have interstitial fibrosis, global sclerosis, and lower eGFR at presentation than C4d-ve patients. C4d+ve was associated with increased risk of clinically significant kidney outcomes (Kidney Failure or eGFR decline >50% or Kidney Failure alone) even after adjustments for standard clinical factors and the revised Oxford Score. Patients with diffuse staining pattern did not have different clinicopathological characteristics compared to those with a focal pattern. On the other hand, patients with a global pattern had lower initial eGFR, and were more likely to have segmental sclerosis and interstitial fibrosis than those with segmental C4d staining pattern. We did not detect differences in kidney outcomes between different staining patterns.

Previous studies have shown that mesangial C4d deposits were associated with more advanced kidney histology or decreased kidney function at the time of biopsy [11, 12, 19–22]. Similar to our study, tubulointersitial fibrosis (T1/T2) lesions has been the most consistent feature associated with mesangial C4d deposits [12, 19–22]. However, this association may not be observed in very small studies or studies which consisted only of patients with early stage disease [13, 14, 23]. The associations with other Oxford score indices such segmental (S1) lesions [12], or mesangial hypercellularity (M1) [22] have been variable. Endothelial hypercellularity (E1) lesions and/or crescents (C or C1/C2) lesions, have not been shown to be associated with C4d staining [11, 12, 14, 19]. Although not part of the Oxford Classification, correlations of C4d staining with global sclerosis has been shown in several studies including our study [11, 21, 22]. Despite IgAN being quite common, there has been few studies in East or Southeast Asians adults. In one study of 23 Korean patients, glomerular C4d staining was associated with albuminuria, but the study was too small to detect other correlations [23].

Most studies that have evaluated the value of C4d deposits as a predictor of long term outcome have been in European adults. In a multicenter retrospective study from Spain, Espinosa *et al* found that kidney survival at 20 years was 28% in C4d+ve patients versus 85% in C4d-ve patients, and C4d+ve was a predictor of Kidney Failure independent of other clinical predictors or the Oxford Score [12]. In a study of 74 patients from Portugal, Faria *et al* found that C4d+ve was an independent positive predictor for 50% GFR decline or Kidney Failure [19]. Most of the above studies included patients with a broad range of GFR. More recently, prognostic value of C4d deposits was evaluated in Spanish adults or Brazilian children with early disease with preserved GFR [13, 14]. In these studies, C4d +ve was predictive of long term kidney outcome independent of other clinicopathological features.

In previous studies, the criteria used to define C4d positivity varied widely ranging from 25% to 75% of positive glomeruli [11, 12, 19, 21–23]. The proportion of patients classified as C4d+ve ranged from 33% to 56%. We used the definition of at least one positive glomerulus for positive C4d staining. This resulted in a higher proportion of IgAN patients (67.5%) being positive in our cohort. Segara *et al* used the same criteria as ours in Spanish subjects, and also found that C4d+ve was an independent predictor of kidney outcome [13]. The prevalence of C4d+ve biopsies was only 20% in their study presumably because the patients all had early disease with eGFR above 80ml/min/1.73m^2^.

Few studies have reported the impact of C4d staining pattern on outcomes. The prevalence of diffuse C4d staining (41.7% of all IgAN patients) in our study is similar to other studies [11, 19, 22, 23]. Because many studies used above 50% of glomeruli being stained positive as the criteria for C4d positivity, only patients with diffuse staining were included in these studies, and patients with focal disease would have been classified as negative. The significance of the proportion of glomeruli affected on outcomes is uncertain. In one small study, the percentage of glomeruli positive for C4d correlated with the degree of proteinuria, serum creatinine and degree of tubulointersitial fibrosis at baseline [21]. We did not show any differences between clinicopathological parameters or impact on outcomes between diffuse and focal disease. Similarly, a study of Spanish patients with early disease, the percentage of affected glomeruli did not correlate with any clinical biochemical, or histopathologic variables [13].

There is limited information on the impact of global versus segmental distribution of C4d staining. In two series from Turkey, the prevalence of segmental lesions as a proportion of all C4d+ve glomeruli was about 80% [20, 22], whereas another study from Spain reported a prevalence of 47%, which was similar to our study [11]. None of the studies evaluated the patient characteristics with different patterns. We found that patients with a global pattern had lower GFR and higher S1 and T1/T2 lesion although we did not demonstrate differences in long term outcomes between the 2 patterns. No previous study had examined the effects of certain combinations (focal versus diffuse and segmental versus global) of patterns on long-term outcome. We did not detect any differences in outcome between different combinations in this study, although the numbers in each group were rather small.

The exact mechanisms that lead to C4d deposition in IgAN or that accounts for a worse prognosis for C4d deposition are presently unknown. The fact that some patients who were previously negative may acquire positive C4d staining suggest that the deposition of C4d may be a dynamic process [13]. IgA1-induced activation of the lectin pathway, which likely underlies C4d deposition, could specifically recruit other amplifying mechanisms to render complement system activation more aggressive [9, 24, 25]. However, although there may be several candidates, a consistent trigger for the lectin pathway activation in IgAN has been not been identified [6]. C4d deposition, even in a limited number of glomeruli, is likely an indicator of more severe, immunologically active disease compared to those without C4d. The situation may be akin to antibody mediated rejection in the kidney allograft, in which C4d positive staining even in less than 10% of glomeruli is associated with worse graft outcome [26]. Once the complement activation pathways have been activated, differences between focal and diffuse patterns may not be observed, because the underlying processes are similar. A kidney biopsy only samples a tiny part of the kidney. C4d deposits may be patchy. Whether C4d deposition in a particular biopsy sample affects above or below 50% of glomeruli could be a function of the part of the kidney that was sampled. Therefore, a difference in outcomes between focal and diffuse patterns may not be detectable because the biopsied samples do not fully represent the whole kidney involvement. The lack of differences in outcomes between focal pattern and diffuse disease may be similar to lupus nephritis in which the clinical outcomes of class III (<50% of glomeruli involved) and class IV (≥ 50% glomeruli involved) are similar [27].

To our knowledge, this large cohort study is the first study to evaluate the risk of C4d deposits in Asian adults with IgAN using clinically important end points. The high multivariate adjusted hazard ratio of 3 is in keeping other studies [12, 13, 19]. Our study provide further supports the role of mesangial C4d deposit as a biomarker for progression of IgA that may useful in both Caucasians and Asians. Interpretation of C4d staining is relatively specific with a low possibility of false positive results [28]. Moreover, staining for C4d is a relatively inexpensive, easy to perform, and therefore, could be applicable for routine use. At present, it is unclear which subset of patients would gain most benefit from C4d staining. Our study suggests that knowledge of C4d status may be useful in all risk groups, but largest difference in outcome between Cd4+ve and Cd4-ve patients may be seen in the group with highest risk for progression based on standard clinical and MEST+C parameters, although additional studies are needed to confirm this. Our definition of C4d+ve patients required only one positive glomerular staining which is less stringent than most previous studies. This study is the only study to compare hard outcomes in patients with diffuse disease with those with focal disease, who would not have been classified as C4d+ve in some series. The lack of differences in clinical presentation and long term outcomes between diffuse and focal disease imply a prognostic value for lower proportion of positive glomeruli, which is supported by results from another recent study [13]. Although we found some differences in clinical characteristics between those with segmental and global pattern, there were no differences in long-term outcome. More studies will be needed to evaluate this issue. Our findings suggests that even one nonsclerotic glomerulus positive for C4d in mesangial cells involving more than 10% of glomerulus regardless of pattern of distribution might be considered to be C4d+ve, but additional studies in other populations will be required before this definition could be used in routine clinical practice.

There are several limitations. Our study is retrospective. Patients were treated according to individual physicians’ preference on the basis of different clinical guidelines available at the time of presentation. The indications for treatment and the therapeutic regimen were not consistent over time. Second, the numbers of patients reaching kidney failure were rather small. Therefore, we used as our primary outcome, a composite outcome of kidney failure and/or 50% reduction in eGFR, which may be less robust than using kidney failure alone. However, since disease progression to kidney failure typically takes many years, combined kidney failure and/or 50% reduction in eGFR has been widely used as a primary outcome in many large studies of IgAN [3, 29]. The finding that C4d was independently associated with the development of kidney failure as a secondary outcome in this study supports an important role for C4d staining in disease prognosis. Third, some C4d positive patients have been misclassified as C4d negative because of sampling variations since C4d deposits only affecting a few glomeruli may have been missed. Finally, the classification of patients as C4d+ve or C4d-ve was based on kidney biopsy performed at diagnosis. It is possible that as the disease progresses, more patients develop positive staining.

## Conclusions

Positive mesangial C4d deposits increased the risk of developing adverse kidney outcomes in Asian patients with IgAN independent from other traditional predictors similar to other studies in Western populations. Different staining patterns did not appear to affect prognosis. Given that this is a relatively inexpensive test, C4d staining may be fully evaluated for routine use in IgAN. This study suggests that even one positive glomeruli might increase the risk of kidney failure or function decline, but more studies are needed to confirm this finding.

## Supporting information

S1 Fig Kaplan-Meier analysis of focal versus diffuse mesangial C4d staining pattern on primary outcome (Kidney Failure or ΔGFR50%). (TIF)

S2 Fig Kaplan-Meier analysis of segmental versus global mesangial C4d staining on primary outcome (Kidney Failure or ΔGFR50%). (TIF)

S1 File (XLSX)
