# Supplementary figures and images for: Distribution pattern of mesangial C4d deposits as predictor of kidney failure in IgA nephropathy
Source: PLoS One. 2021 Jun 3;16(6):e0252638. doi: 10.1371/journal.pone.0252638 (PMC8174712; doi:10.1371/journal.pone.0252638)

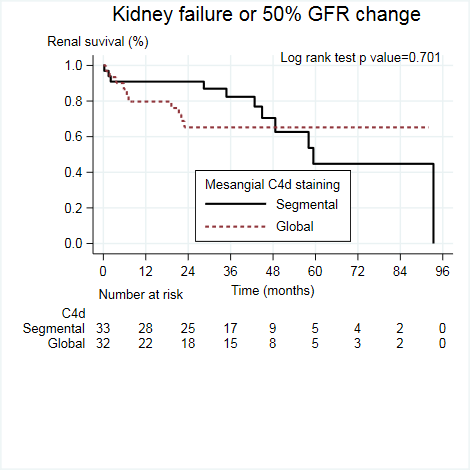

Supplement: S1 Fig — (TIF) [file pone.0252638.s001.tif]

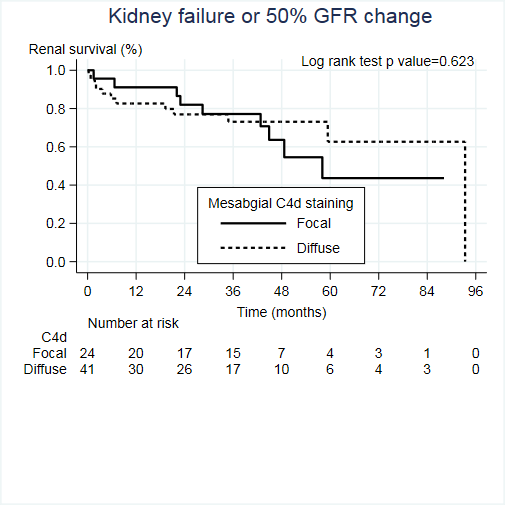

Supplement: S2 Fig — (TIF) [file pone.0252638.s002.tif]
